# Clinical chorioamnionitis: where do we stand now?

**DOI:** 10.3389/fmed.2023.1191254

**Published:** 2023-05-24

**Authors:** David Lukanović, Marija Batkoska, Gorazd Kavšek, Mirjam Druškovič

**Affiliations:** ^1^Division of Obstetrics and Gynecology, Ljubljana University Medical Center, Ljubljana, Slovenia; ^2^Department of Gynecology and Obstetrics, Faculty of Medicine, University of Ljubljana, Ljubljana, Slovenia; ^3^Division of Obstetrics and Gynecology, Department of Perinatology, Ljubljana University Medical Center, Ljubljana, Slovenia

**Keywords:** chorioamnionitis, intrauterine infection, pregnancy, outcome, management, labor

## Abstract

Intraamniotic infection is an infection resulting in the inflammation of any combination of the amniotic fluid, the placenta, the fetus itself, the fetal membranes, umbilical cord, or the decidua. In the past, an infection of the amnion and chorion or both was dubbed *chorioamnionitis*. In 2015, a proposal was made by an expert panel that, instead of *clinical chorioamnionitis*, the name *intrauterine inflammation or infection or both* be used, abbreviated as *Triple I* or simply *IAI*. However, the abbreviation *IAI* did not gain popularity, and this article uses the term *chorioamnionitis*. Chorioamnionitis may arise prior to, during, or following labor. It can present as a chronic, subacute, or acute infection. Its clinical presentation is generally referred to as *acute chorioamnionitis*. The treatment of chorioamnionitis varies widely across the world due to different bacterial causes and the absence of sufficient evidence to support a specific treatment regimen. There are limited randomized controlled trials that have evaluated the superiority of antibiotic regimens for treating amniotic infections during labor. This lack of evidence-based treatment suggests that the current choice of antibiotics is based on limitations in existing research, rather than absolute science. Chorioamnionitis cannot be cured by antibiotic therapy alone without delivery, and therefore it is necessary to make a decision according to the guidelines for induction of labor or acceleration of delivery. When a diagnosis is suspected or established, it is therefore necessary to apply broad-spectrum antibiotics according to the protocol used by each country, and to continue with them until delivery. A commonly recommended first-line treatment for chorioamnionitis is a simple regimen consisting of amoxicillin or ampicillin and once-daily gentamicin. Available information is not sufficient to indicate the best antimicrobial regimen to treat this obstetric condition. However, the evidence that is currently available suggests that patients with clinical chorioamnionitis, primarily women with a gestational age of 34 weeks or more and those in labor, should receive treatment with this regime. However, antibiotic preferences may vary based on local policy, clinician experience and knowledge, bacterial reasons for the infection, antimicrobial resistance patterns, maternal allergies, and drug availability.

## 1. Introduction

Intraamniotic infection is an infection resulting in the inflammation of any combination of the amniotic fluid, the placenta, the fetus itself, the fetal membranes, the umbilical cord, or the decidua (1). Previously, an infection of the amnion and chorion or both was dubbed *chorioamnionitis*. In 2015, a proposal was made by an expert panel that, instead of *clinical chorioamnionitis*, the name *intrauterine inflammation or infection or both* be used, abbreviated as *Triple I* or simply *IAI* (2). However, the abbreviation *IAI* did not gain popularity, and this article uses the term *chorioamnionitis* (3, 4).

Pathologists use the name *histologic chorioamnionitis* to refer to an inflammation that lacks typical microbiological or clinical findings connected with acute infection, which adds to the complexity of terms (1, 3). Diagnoses of clinical and histologic chorioamnionitis overlap considerably, but they are not always concurrent. The reason for this may lie in subclinical chorioamnionitis, which can be identified based on examination of the placenta but may still not be clinically diagnosed, or because non-specific clinical signs are used to diagnose clinical chorioamnionitis (5).

Chorioamnionitis can arise prior to, during, or following labor. It may be chronic, subacute, or acute. Its clinical presentation is generally referred to as *acute chorioamnionitis*. Chorioamnionitis is most often linked with premature labor, prolonged membrane ruptures, prolonged labor, smoking, meconium-stained amniotic fluid, nulliparous pregnancy, multiple vaginal exams following rupture of membranes, and identified viral or bacterial infections. Chorioamnionitis can also arise at term and appear in women without previous infections. Chorioamnionitis can result in morbidity and mortality in the mother and the newborn if not treated in time and appropriately. The morbidity and mortality of the newborn increase with early pregnancy. It has been shown that antibiotic treatment decreases the frequency and severity of chorioamnionitis in both mothers and newborns (1, 3, 6).

The clinical picture of chorioamnionitis is presented below with an emphasis on current treatment and the use of antibiotics. This article presents various guidelines used by various countries and identifies differences in the treatment approaches.

### 1.1. Epidemiology

A systematic review performed by Woodd et al. (7) estimated that clinical chorioamnionitis occurs in 3.9% of all puerperae and that it is the most common infection during labor (7, 8). The incidence of chorioamnionitis varies greatly between studies (9). This difference is the result of multiple factors, especially variations in research methodology (higher rates are reported in prospective studies in comparison to retrospective studies), variations in the distribution of risk factors among the populations investigated, the application of various diagnostic criteria (e.g., clinical compared to histologic ones: the incidence of histologic chorioamnionitis is much greater), and changes in obstetric practice (3, 6). Incidence also differs between preterm labor (prior to 37 weeks’ gestation) and labor at term (37 weeks or more). In women with preterm labor or that have preterm premature rupture of membranes (PPROM), the incidence is 40–70% (10–12). In deliveries occurring from 21 to 24 weeks of gestation, it is possible to find histologic chorioamnionitis in over 94% of cases (9). In term pregnancies, clinical chorioamnionitis was diagnosed in roughly 1–3% of cases with membranes that were intact and 6–10% of cases having preterm rupture of membranes (PROM) (11, 12).

### 1.2. Pathogenesis and microbiology

The origin of chorioamnionitis is often polymicrobial; it involves both anaerobic and aerobic bacteria, and it often arises from the vaginal flora (3). It mostly occurs through bacterial invasion ascending from the lower genital tract into the amniotic cavity, which is usually sterile. Ascending infections, progression, and clearance may be linked to vaginal dysbiosis, according to recent research (13). Although microbiome diversity is typically indicative of good health in most areas of the body, the vaginal microbiome’s health is instead tied to low microbial heterogeneity and the predominance of *Lactobacillus* spp. This genus produces enzymes capable of glycogen fermentation, leading to the production of substantial amounts of lactic acid. The subsequent low pH protects the cellular metabolic function of the cervix and vagina and impedes the growth of potentially pathogenic species. In addition, genetic factors may impact mucosal immunity and microbiome diversity (13).

**FIGURE 1:**
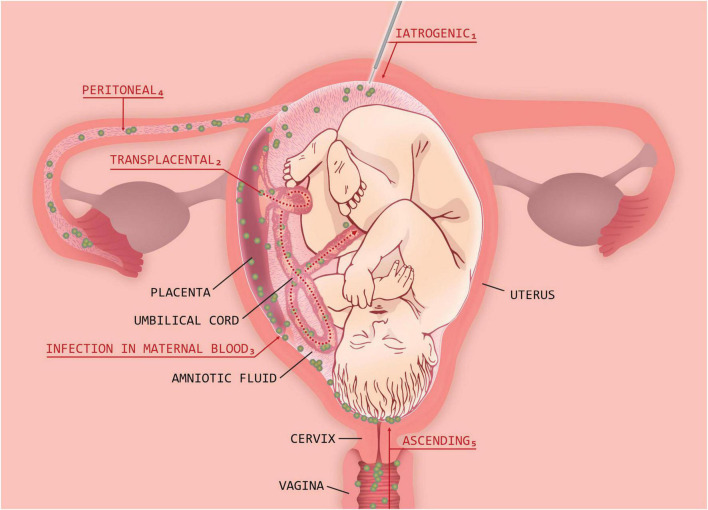
Potential routes of intrauterine infection/chorioamnionitis.

In rare cases, it is also possible for intraamniotic infection to occur following invasive procedures such as amniocentesis or chorionic villus sampling or via a hematogenous route that is secondary to maternal systemic infection—for example, with *Staphylococcus aureus* or *Listeria monocytogenes*. Potential routes of intrauterine infection/chorioamnionitis are shown in the Figure 1. Most cases of chorioamnionitis detected and managed by obstetricians are noted at term, and clinically apparent intraamniotic chorioamnionitis complicates only 2–5% of these deliveries (1). More recent information shows that the relative risk of chorioamnionitis and neonatal infection increases from 40 weeks of gestation onward (1, 9).

The pathophysiology of chorioamnionitis is highly complicated. One of the most important cytokines responsible for the inflammation response is tumor necrosis factor-alpha (TNF-α), which is a multifunctional Th1 cytokine and is produced by macrophages during inflammation. Cytokine homeostasis allows for embryo implantation and normal pregnancy outcomes. In the early stages of normal pregnancy, Th1 pro-inflammatory cytokines are necessary for the stimulation of new vessels for successful embryo implantation. However, prolonged exposure to Th1 cytokines may result in a cell-mediated immune response, which is harmful to the fetus and may cause spontaneous abortion or preterm birth (14). Recent studies have demonstrated an important correlation between TNF-α and the endocannabinoid/endovanilloid (EC/EV) system in preterm deliveries. Torella et al. found a link between the stimulation of cannabinoid receptor type 1 (CB1) and the antagonism of the transient receptor potential vanilloid 1 (TRPV1) channel in the placenta, which could be used in preterm birth prevention through selected molecules (15).

The bacteria involved in chorioamnionitis may vary by location and specific population. Bacteria that are commonly identified in chorioamnionitis are group B streptococcus (GBS), *Bacteroides* spp. *Escherichia coli*, *Gardnerella vaginalis*, *Mycoplasma pneumoniae*, and *Ureaplasma* spp. *Candida* and its subspecies are defined as risk factors linked with chorioamnionitis, which leads to preterm birth and neonatal infections. Studies have shown that in young persons with sexually transmitted infections trichomoniasis constitutes a risk for developing chorioamnionitis. Even though chorioamnionitis represents a risk factor for vertical transmission during pregnancy, HIV status of the mother does not represent a risk factor with regard to chorioamnionitis (1, 2, 4, 6, 8, 16).

In addition to the aforementioned pathogens, there are many that can activate the inflammation cascade but are not often mentioned. Zika virus infection, which can lead to chorioamnionitis, is possible in pregnancies in high-risk areas. It mainly crosses the placenta in the first trimester and therefore is rarely considered in pregnancy. Ascending vaginal infection is also possible (17).

Infections in non-genital sites such as pneumonia and periodontal infection are linked to preterm labor. Oral infections are considered a contributing factor to preterm labor incidence because research has found that commensal bacterial species from the oral cavity can spread to the fetoplacental unit of women with term gestation and adverse pregnancy outcomes. Adverse pregnancy outcomes have been strongly associated with certain microbes, including *Fusobacterium nucleatum*, *Campylobacter rectus*, *Porphyromonas gingivalis*, and *Bergeyella* spp. (18).

### 1.3. Risk factors

The most important risk factors for chorioamnionitis are prolonged labor and the time from the spontaneous rupture of the fetal membranes to birth. Increased risk for the development of chorioamnionitis is also associated with the following (1, 3, 19):

• GBS infection during pregnancy, sexually transmitted diseases, bacterial vaginosis;
• Multiple digital vaginal examinations during labor (especially post rupture of membranes);
• Digital examinations instead of speculum examinations of pregnant women with PPROM;
• Cervical insufficiency;
• Intracervical balloon catheter to help the cervix ripen faster or induce labor;
• Meconium-stained amniotic fluid;
• Alcohol abuse and smoking during pregnancy;
• Pregnancy after IVF-ET;
• Chorioamnionitis in previous pregnancies.

## 2. Signs and symptoms

Maternal fever over 38°C and fetal tachycardia even before the onset of fever predominate in the clinical picture of chorioamnionitis in pregnant women. In laboratory blood test results, the diagnosis is confirmed by elevated inflammation indicators and a typically raised white blood cell count that has a left shift; in addition, purulent amniotic fluid and uterine tenderness on palpation are also typical (20).

The clinical picture is often non-specific and may include one or more of the following symptoms (1, 2, 6):

• Fever;
• Leukocytosis in the mother exceeding 15,000/mm^3^;
• Tachycardia in the mother exceeding 100/min;
• Tachycardia in the fetus exceeding 160/min;
• Uterine tenderness on palpation;
• Bacteremia (most common when chorioamnionitis is associated with a GBS or *E. coli* infection;
• Purulent amniotic fluid.

Chorioamnionitis can have a subclinical manifestation, which is defined as not presenting with the clinical image described above. A subclinical infection can present as preterm labor with intact fetal membranes or as PPROM (4). We approach pregnant women with suspected chorioamnionitis in stages. The patient history should start with the age of the mother, gestational age, parity, major characteristics of the pregnancy including any difficulties, and the history of sexually transmitted infections, urinary tract infections, and other illnesses. The status of the fetal membranes is important: whether there has been a rupture or whether the membranes are preserved, and whether meconium-stained amniotic fluid is present. We measure the basic vital functions of a pregnant woman (temperature, blood pressure, pulse, and oxygen saturation). The physical examination should be thorough; it should also include a complete physical assessment, including an examination of the abdomen and vagina, and ultrasound examination of the uterus and fetus. Upon admission and suspicion of chorioamnionitis, it is necessary to take a swab of the vagina for pathogenic bacteria. The initial assessment of chorioamnionitis thus includes a complete maternal and fetal clinical assessment. A blood count (for leukocytosis) is routine for suspected infection, but recent studies suggest that leukocytosis in pregnant women with PPROM on admission does not confirm microbial invasion into the amniotic cavity or inflammation. Bacterial cultures taken via vaginal or cervical swab do not correlate with infection secondary to chorioamnionitis (1, 3, 4, 6).

Chorioamnionitis can be diagnosed using amniotic fluid culture or Gram staining—or both of these methods plus biochemical analysis—but in most puerperae such a diagnosis is primarily based on clinical evaluation of symptoms and signs. According to the panel of maternal and neonatal experts (the American College of Obstetricians and Gynecologists; the American Academy of Pediatrics; the Eunice Kennedy Shriver National Institute of Child Health and Human Development, NICHD; and the Society for Maternal Fetal Medicine), chorioamnionitis/intraamniotic infection is divided into three different categories (1, 2): (1) maternal fever that is isolated (not Triple I), (2) suspected Triple I, and (3) confirmed Triple I. The individual categories are explained in detail and presented in Table 1.

**TABLE 1:** Features of isolated maternal fever and Triple I with classification.

Category	Features and comments
Isolated maternal fever (“documented” fever)	Maternal oral temperature ≥ 39.0°C (102.2°F) on any one occasion is documented fever. If the oral temperature is 38.0°C (100.4°F) to 38.9°C (102.02°F), repeat the measurement in 30 min; if the repeat value remains at least 38.0°C (100.4°F), it is documented fever.
Suspected Triple I	Fever without a clear source plus any of the following: Baseline fetal tachycardia (greater than 160 beats per min for 10 min or longer, excluding accelerations, decelerations, and periods of marked variability) Maternal white blood cell count greater than 15,000 per mm^3^ in the absence of corticosteroids Definite purulent fluid from the cervical os
Confirmed Triple I	All of the above plus: Amniocentesis: proven infection through a positive Gram stain Low glucose or positive amniotic fluid culture Placental pathology revealing diagnostic features of infection

Adapted from Higgins et al. (2).

Markers before delivery combined with clinical manifestations and gestational age are useful for guiding management for the mother. Confirmation of chorioamnionitis using antenatal markers is not routinely required in women at term that are showing progress toward delivery. However, for women experiencing preterm labor that are undergoing evaluation for tocolysis or using corticosteroids, biomarker assessment may have some value in confirming a diagnosis of chorioamnionitis (21). In clinics where amniocentesis is carried out to ascertain chorioamnionitis, the lab tests used for clinical management include lactate dehydrogenase (LDH) activity, glucose concentration, WBC and RBC counts, bacterial cultures, and Gram stain. The results of cultures are not usually available promptly enough for making decisions. Consequently, physicians are forced to rely on other analyses, the turnaround times for which are only hours. Unfortunately, these tests (glucose, LDH, WBC count, and Gram stain) sometimes do not agree in confirming or ruling out chorioamnionitis; consequently, interpreting the results of these tests may not be simple. Studies examining biomarkers of chorioamnionitis are complicated by the absence of a gold standard for diagnosis (2). The various biomarkers that have undergone evaluation are not ideal; that is because their accuracy is insufficient for defining a particular threshold or due to the invasive character of amniocentesis. Some studies have noted the promise of IL-6 as an intrauterine inflammation marker (22, 23). Nonetheless, current information remains debated. A Cochrane review recently showed that when AF analysis is used to exclude chorioamnionitis in women with PPROM it suffers from low evidence quality (21, 24).

Chorioamnionitis can range from mild to severe. Histopathological results agreeing with inflammation may also be found in placentas during normal pregnancies (25). In cases of chorioamnionitis, the fetal membranes can appear normal or they may show signs of infection. The amniotic fluid may be clear or opaque. On histological examination, neutrophilic infiltration is present in the decidua and, in more serious cases, there are microabscesses. A publication by Faro et al. (26) demonstrated that neutrophils found in the amniotic fluid mostly originate from the fetus. In cases of extreme prematurity, neutrophils from the mother and fetus are more often found in the amniotic fluid in chorioamnionitis (6, 25). The mother’s immune response to chorioamnionitis causes the chorioamnion to undergo neutrophilic inflammation, and the fetal immune response causes neutrophilic inflammation affecting the umbilical cord (i.e., funisitis) and/or the chorionic plate’s fetal vessels (i.e., chorionic vasculitis). Chorioamnionitis has a greater frequency than funisitis and occurs in nearly 100% of cases of funisitis, whereas funisitis occurs in 60% of cases of chorioamnionitis (1, 6, 25). The histological criteria for chorioamnionitis are shown in Tables 2, 3. A histological diagnosis of chorioamnionitis can be made even without any clinical signs and symptoms of infection or positive cultures from the placenta, the fetal membranes, or the amniotic fluid. In such cases, inflammatory developments in the membranes may be caused by non-contagious infections (hypoxic injuries, meconium, trauma, or allergens). Yet another explanation of negative cultures is the fact that cultures are not sensitive to certain organisms, including genital mycoplasmas, which are the organisms that are most commonly linked with chorioamnionitis. A role is also played by antibiotic therapy before delivery. In a study by Queiros da Mota et al. (27), the histological and bacteriological results matched in roughly 70% of the total 376 placentas investigated (27). If the chorioamnionitis diagnosis was determined on the basis of a positive microbiological culture of the amniotic fluid, histology showed 83–100% sensitivity and 23–52% specificity (28).

**TABLE 2:** Grading and staging of maternal inflammatory response in acute chorioamnionitis.

Category	Terminology	Definition
Stage 1 (early)	Acute subchorionitis or chorionitis	Acute inflammation limited to the subchorionic space or membranous trophoblast, not extending into fibrous chorion
Stage 2 (intermediate)	Acute chorioamnionitis	Diffuse or patchy acute inflammation of the fibrous chorion with or without amnion
Stage 3 (advanced)	Necrotizing chorioamnionitis	Necrotizing acute inflammation of the chorion and amnion characterized by neutrophil karyorrhexis, amniocyte necrosis, with or without amnion basement membrane thickening or hypereosinophilia
Grade 1 (mild–moderate)		Scattered neutrophils
Grade 2 (severe)	Severe acute chorioamnionitis with or without subchorionic micro-abscesses	Confluent neutrophils

Adapted from Redline et al. (57) and Heerema-McKenney et al. (58).

**TABLE 3:** Grading and staging of fetal inflammatory response in acute chorioamnionitis.

Category	Terminology	Definition
Stage 1	Chorionic vasculitis or umbilical phlebitis	Involves umbilical vein (with or without chorionic plate vessels)
Stage 2	Umbilical vasculitis	Involves one or both umbilical arteries with or without scattered neutrophils in Wharton’s jelly
Stage 3	Necrotizing funisitis, concentric umbilical perivasculitis	Concentric acute inflammation with karyorrhectic debris, eosinophilia of matrix with or without mineralization
Grade 1 (not severe as defined)	No additional terminology	Scattered neutrophils beneath endothelium or in vascular smooth muscle, not confluent
Grade 2 (severe)	With severe fetal inflammatory response	Confluent neutrophils beneath endothelium or in vascular smooth muscle with attenuation of vascular smooth muscle
Other	Peripheral funisitis	Focal aggregates of neutrophils at umbilical cord surface

Adapted from Redline et al. (57) and Heerema-McKenney et al. (58).

## 3. Differential diagnosis

The majority of clinical signs linked with chorioamnionitis are not specific; in a pregnant woman, fever may be associated with dehydration, use of prostaglandins for cervical ripening or induction of labor, or epidural analgesia. Maternal tachycardia can be physiological or associated with pain, epidural analgesia, or medication. Leukocytosis in a pregnant woman occurs both during childbirth and during antenatal treatment with corticosteroids and also in infections that are not chorioamnionitis. Tachycardia in the fetus may be associated with hypoxemia in the fetus, fever of any etiology in the mother, or the passage of certain drugs via the placenta (1, 3, 4, 6).

Differential diagnosis in pregnant women with clinical signs of chorioamnionitis includes delivery, placental abruption, and other infections. Labor may be linked with fever (if the puerpera has had epidural analgesia), tachycardia in the mother, leukocytosis, and a uterus tender to palpation. Clinical chorioamnionitis is difficult to diagnose in puerperae with epidural analgesia because fever is frequent and can be associated with the anesthetic. Moreover, epidural anesthesia hides the sensitivity of the uterus and may cause tachycardia in the mother or fetus. On the other hand, a small abruption can result in increased uterine sensitivity and tachycardia in the mother, but it is usually linked with absence of fever and vaginal bleeding. Other, extrauterine infections linked with abdominal pain (not necessarily with labor) and fever and including pyelonephritis, viral respiratory infections, appendicitis, pneumonia, and COVID-19 are also considered in the differential diagnosis. Such infections can result in tachycardia and leukocytosis in the mother as well as tachycardia in the fetus. Nonetheless, they can generally be distinguished from chorioamnionitis based on clinical presentation (e.g., gastrointestinal or respiratory symptoms indicate an extrauterine cause of fever) and through lab tests (e.g., urinalysis) (1, 3, 4, 6, 21).

## 4. Complications

Exposure to chorioamnionitis increases the risk of an adverse pregnancy outcome by 2- to 3.5-fold, independent of the duration of infection. Sepsis in the mother and neonate are leading causes of death globally. According to the literature, between 2003 and 2009, sepsis resulted in 10.7% of maternal deaths worldwide (25). Even though maternal death as a result of sepsis is more frequent in less-developed countries, it is a growing problem in some developed countries, such as the United States (5).

At present, chorioamnionitis serves as a primary risk factor to identify infants at risk of early-onset neonatal sepsis (EONS). However, if newborns are overexposed to broad-spectrum antibiotics before EONS is excluded, or for “presumed” EONS without a definitive diagnosis, this has the potential to cause short- and long-term adverse effects, including increased risk of necrotizing enterocolitis and mortality (5, 29–31). Late-onset neonatal sepsis (LONS) is challenging to diagnose because the clinical signs are non-specific, and no risk stratification tools are available as a guide toward a threshold for when diagnostic tests should be ordered or antibiotics started. It is necessary to quantify the risk of both EONS and LONS following exposure to chorioamnionitis to assist in clinical decision-making (5). Adverse fetal/neonatal outcomes include perinatal death, asphyxia, pneumonia, meningitis, intraventricular hemorrhage (IVH), respiratory distress syndrome, bronchopulmonary dysplasia in preterm infants, and long-term disability, including cerebral palsy, and morbidity associated with prematurity (6). Among pregnancies with clinical chorioamnionitis, 6% of newborns were diagnosed with EONS. Among neonates with EONS, up to 40% of cases were associated with clinical chorioamnionitis (5). Premature infants have a greater rate of short-term complications from chorioamnionitis compared to full-term infants. They face increased rates of respiratory distress (62 versus 35% in preterm versus full-term infants), neonatal sepsis (28 versus 6%), perinatal death (25 versus 6%), third- or fourth-degree IVH (24 versus 8%), and pneumonia (20 versus 3%) (32, 33).

In a recent meta-analysis and systematic review, Villamor-Martinez et al. (34) concluded that the immaturity of an infant is key in the morbidity linked with very preterm or extremely preterm birth; however, the pathological processes resulting in preterm birth can also affect the outcome. Their data indicate that, if infection or inflammation serve as triggers for preterm birth, such infants are more inclined to develop sepsis not only directly following birth, but also during the first weeks of their lives. The link between chorioamnionitis and EONS does not appear to be connected with gestational age, whereas the lower gestational age of infants exposed to chorioamnionitis reduced the effect size of the link between LONS and chorioamnionitis. According to Villamor-Martinez et al. (34), chorioamnionitis may start the immunomodulatory sequence that leads to LONS, but it may also modify the exposure rate to other stimuli, including antenatal and post-natal antibiotics and corticosteroids, invasive therapies, lung damage, patent ductus arteriosus, or necrotizing enterocolitis, which result in greater vulnerability of very preterm or extremely preterm infants to sepsis.

On the other hand, chorioamnionitis may cause serious complications in the mother, including adult respiratory distress syndrome (ARDS), intensive care unit (ICU) admission, the need for a hysterectomy, post-partum endometritis, post-partum hemorrhage, prolonged labor, sepsis, wound infection, and, in unusual cases, maternal mortality (2). Women experiencing clinical chorioamnionitis are at increased risk of uterine atony, blood transfusion, and postpartum hemorrhage compared to women that do not have clinical chorioamnionitis. Increased postpartum hemorrhage and uterine atony seem to be related directly to myometrial contractility impairment as a result of intraamniotic inflammation or infection (4). Beck et al. (5) also pointed out that, although there are large numbers of studies evaluating neonatal sepsis, most of them were without maternal characteristics, and so their systematic review was unable to conclude if risk changes as a result of baseline clinical and demographic characteristics. Moreover, it was not possible to evaluate the association between maternal sepsis and chorioamnionitis due to the lack of publications available (5). Taking into account the limitations of published articles, further research on this topic is especially important due to high maternal mortality rates, especially in the United States in comparison to similar developed countries.

## 5. Management

The treatment of chorioamnionitis varies widely across the world due to different bacterial causes and the absence of sufficient evidence to support a specific treatment regimen. There are limited randomized controlled trials (RCTs) that have evaluated the superiority of antibiotic regimens for treating amniotic infections during labor. This lack of evidence-based treatment suggests that the current choice of antibiotics is based on limitations in existing research, rather than absolute science.

Even though chorioamnionitis is linked to preterm labor and delivery, there is no evidence to support antibiotics being routinely administered to women in preterm labor if they have intact membranes and there are no overt signs of infection. In fact, such prophylaxis may worsen outcomes (35). However, intrapartum antibiotics have been effective in preventing EONS and have significantly reduced its incidence in countries where they are used, regardless of the regimen used (35).

**FIGURE 2:**
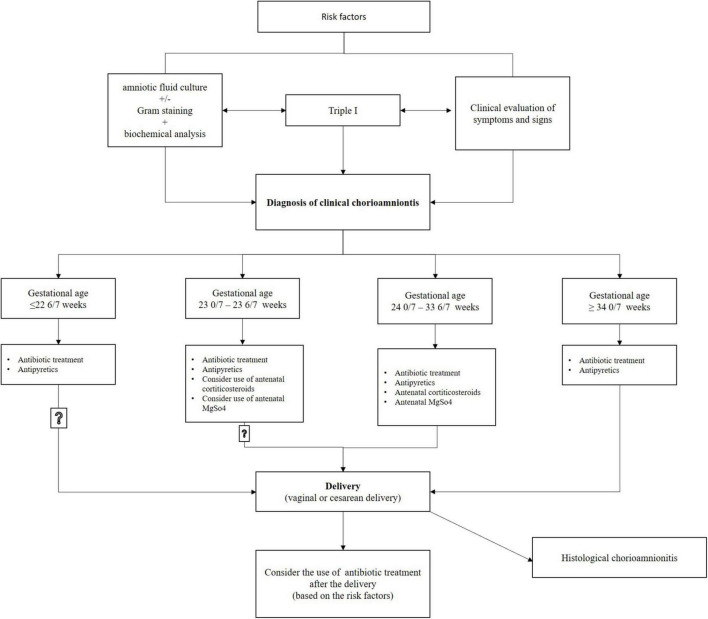
Management of clinical chorioamnionitis.

Pregnant women with chorioamnionitis (including suspected or confirmed) should be started on antibiotic therapy, and a decision should be made to induce labor; Figure 2. At present, evidence indicates that administering magnesium sulfate for fetal neuroprotection plus antenatal corticosteroids for fetal lung maturation to patients experiencing clinical chorioamnionitis between gestational ages of 24 0/7 and 33 6/7 weeks, and perhaps also between 23 0/7 and 23 6/7 weeks, provides a generally beneficial effect for the infant.

Nonetheless, there should be no delay of delivery to finish the complete course of magnesium sulfate and corticosteroids (4). Intravenous antibiotic therapy administered to the pregnant woman ensures concentration of antibiotics in the fetus and amniotic fluid 1–1.5 h after infusion, which reduces the risk of serious complications in the mother and fetus, but delivery is necessary for chorioamnionitis to be cured. The effectiveness of antibiotics is limited because bacteria in the amniotic fluid form biofilms that are resistant to antibiotic treatment (1, 4, 6). Immediate induction or acceleration of labor according to the protocol and guidelines is necessary in pregnant women with chorioamnionitis, and cesarean section should only be performed in the presence of obstetric indications. When cesarean delivery is applied with chorioamnionitis, this raises the risk of venous thrombosis, endomyometritis, and wound infection (1, 4, 36).

Antibiotic treatment started due to suspected or confirmed chorioamnionitis should not automatically continue after delivery; the extension of antibiotic treatment should be based on risk factors for endometritis after delivery (1, 36). According to a study by Edwards and Duff (22), there is a lower likelihood of endometritis in postpartum women that delivered vaginally and may not require antibiotic therapy after delivery (1, 22). In postpartum women that delivered by cesarean section, at least one additional antibiotic dose is recommended after delivery (1). However, based on the existence of other maternal risk factors, such as postpartum bacteremia or fever, it can be decided to continue antimicrobial treatment; Figure 2.

### 5.1. Differences in guidelines between countries

According to the American College of Obstetricians and Gynecologists (ACOG), antibiotics should be used whenever chorioamnionitis is suspected or confirmed in the absence of clearly documented overriding risks (1). In addition to antibiotics, the use of antipyretics is also necessary. The guidelines of the National Institute for Health and Care Excellence (NICE) (36) indicate that antibiotics should be administered to a woman with a clinical diagnosis of chorioamnionitis during labor. It is also necessary to give antibiotics to a pregnant woman immediately if an infection is suspected and to continue using them until the baby is born (36).

Many countries follow the ACOG guidelines, including Italy (37), China (38), India (39), and Sweden. Many countries have used the existing recommendations and adjusted them according to their counties’ situation, including antibiotic availability, bacteria resistance, and so on.

**FIGURE 3:**
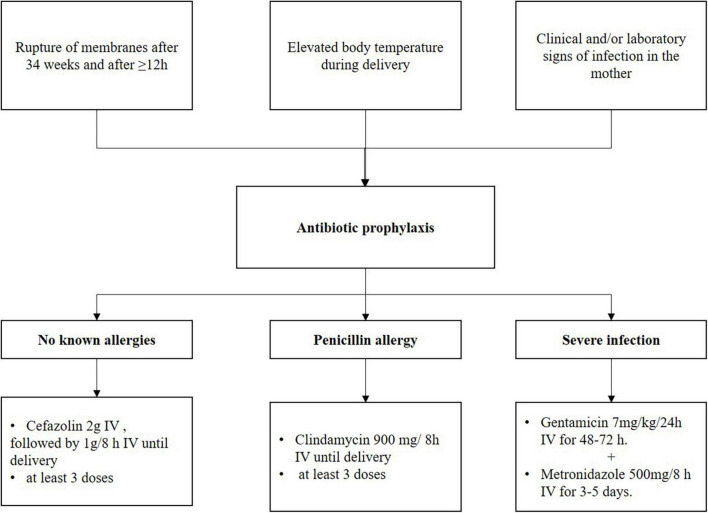
Management of prevention infection in the mother at delivery, the Slovenian recommendations. Adapted from Fabjan Vodušek et al. (40).

The Slovenian guidelines (40) state that antibiotics are used during childbirth to prevent and treat infections in the mother and to prevent infections in newborns; Figure 3. Short-term treatment during childbirth is used to prevent infections with GBS in newborns, to prevent postpartum endometritis, and to prevent or treat infections in the mother (postpartum endometritis, amnionitis, and chorioamnionitis). Antibiotics for preventing infections are administered when there are clinical and/or laboratory signs of inflammation in the mother and/or when the mother has a fever during childbirth. In addition, antibiotics are administered when there is rupture of the fetal membranes after the 34th week of pregnancy lasting for more than 12 h (23).

Table 4 shows various antibiotic regimens for the treatment of chorioamnionitis, including the ACOG and NICE recommendations. The emphasis is on the different regimens in several European countries—Spain (41), France (42), and Slovenia (40)—that have distinctive antibiotic recommendations.

**TABLE 4:** Antibiotic treatment scheme for prevention or treatment of infections in mothers according to ACOG (1), NICE (36), Spanish (41), French (42), and Slovenian (40) guidelines and recommendations.

	ACOG recommendation (1)	NICE recommendation (36)	Spanish recommendation (41)	Slovenian recommendation (40)*	French recommendation (42)
No known allergies	Ampicillin 2 g/6 h IV + Gentamicin 5 mg/kg/24 h IV	Benzylpenicillin + Gentamicin + Metronidazole	Piperacillin-tazobactam 4 g/6 h IV + Clarithromycin 500 mg/12 h PO	Cefazolin 2 g IV, followed by 1 g/8 h IV until delivery (at least 3 doses). The length of cefazolin therapy is adjusted based on laboratory and clinical signs of inflammation.	Amoxicillin 3 g/24 h + Gentamicin 3 mg/kg/24 h in 1 injection
Mild penicillin allergy	Cefazolin 2 g/8 h IV + Gentamicin 5 mg/kg/24 h IV	Cephalosporins sensitive to GBS (e.g., cefotaxime) + Metronidazole	Teicoplanin 600 mg IV 1 dose, after 12 h PO 400 mg/12 h PO for 24 h and then 400 mg PO every 24 h together with Aztreonam 1 g/8 h PO + Clarithromycin 500 mg/12 h PO	Clindamycin 900 mg/8 h IV until delivery or a total of at least 3 doses.	Ceftriaxone 1 g/24 h IV + Gentamicin 3 mg/kg/24 h IV
Severe penicillin allergy	Clindamycin 900 mg/8 h IV or Vancomycin 1 g/12 h IV + Gentamicin 5 mg/kg/24 h IV	Vancomycin + Gentamicin + Metronidazole			Clindamycin 600 mg/6 h IV + Gentamicin 3 mg/kg/24 h IV Or aztreonam (the use of aztreonam requires use of an additional antibiotic active against Gram-positive bacteria)
Severe infection				Gentamicin 7 mg/kg/24 h IV 48–72 h + Metronidazole 500 mg/8 h IV 3–5 days	

*The protocol is currently a subject of revision due to the high level of GBS resistance to clindamycin in Slovenia (37).

## 6. Discussion

A commonly recommended first-line treatment for chorioamnionitis is a simple regimen consisting of amoxicillin or ampicillin and once-daily gentamicin. Even though there is not sufficient information to demonstrate the best antimicrobial regimen for treating chorioamnionitis, the evidence that is currently available shows that women experiencing clinical chorioamnionitis, primarily women with a gestational age of 34 weeks or more and that are in labor, should receive treatment with this regime. These antibiotics are widely available at most facilities around the world and are more cost-effective than other regimens, which may lead to reduced healthcare costs in certain settings (1, 4, 43). Although there is not enough evidence to support one antibiotic over another, most countries prefer a regimen that is easy to administer and follows antibiotic use principles to minimize the emergence of resistant bacterial strains (36). In a recent article, Koucky et al. (44) pointed out that only three recommendations or guidelines [ACOG (1), CNGOF (42), and WHO (43)] addressed this issue, and that there was agreement among them with regard to immediately initiating combination antibiotic therapy and maintaining this for the duration of labor. The guidelines recognized the weakness of evidence to make strong recommendations regarding the duration for which antibiotics should be continued after delivery (44). Maternal GBS colonization is one of the main factors associated with the onset of chorioamnionitis and neonatal infection. As a consequence, a number of professional bodies in the United States, Spain, Australia, and Canada are in favor of universal antenatal screening. In contrast, the New Zealand Medical Association (NZMA) and the Royal College of Obstetricians and Gynaecologists (RCOG) do not support such a policy. The RCOG decision is based on the opinion of the UK National Screening Committee that clear evidence is still lacking regarding the benefits of routine screening for GBS, no fully accurate screening test is available yet, and at 35–37 weeks of gestation the GBS status does not reflect what this status will be at delivery (44–46). On the other hand, the American Academy of Pediatrics stated that broad adoption of the routine antenatal GBS screening policy it recommends has corresponded to an estimated 80% drop in early-onset GBS (44). For example, the proportion of Slovenian pregnant women that are carriers of GBS in the intestine or vagina was determined in two studies (47, 48) and was found to be 17 and 23%, respectively. Pregnant women can be selected for antibiotic prophylaxis: by the presence of perinatal risk factors (premature birth, PPROM, fever, presence of GBS in the urine during pregnancy, or neonatal infection with GBS during a prior pregnancy), in the event of previously established GBS colonization in the third trimester of pregnancy, typically between the 35th and 37th week, or in the case of established perinatal GBS colonization of the mother. Nevertheless, in 2019 the Health Council of Slovenia adopted a proposal to introduce universal screening for GBS in the 35th to 37th week of pregnancy (49).

Moreover, Chatzakis et al. (19) concluded that a number of antibiotics seem to be more effective than using a placebo or no treatment in reducing the rate of chorioamnionitis following PPROM. Nonetheless, none of these are consistently better in comparison to other antibiotics, and the majority are not superior to no treatment or placebo for outcomes besides chorioamnionitis. In their conclusion, the quality of the evidence is low and for some antibiotics it is possibly outdated; moreover, some drugs frequently administered in clinical practice, in particular cephalosporins, have been underrepresented in RCTs. Similar conclusions were pointed out by Conde-Agudelo et al. (4) based on a survey carried out among American obstetricians, which showed broad variation in patterns of practice for managing clinical chorioamnionitis. This survey pointed out that clinicians are using over 25 different regimens of primary antibiotics, and that the duration of postpartum antibiotics ranges from no treatment an all to as much as 48 h of treatment postpartum (4).

It is still unclear if antibiotics ought to be discontinued following birth or continued postpartum. However, if a woman remains symptomatic, extended antibiotic treatment for a minimum of 24 to 48 h after the infection signs and symptoms have subsided may be beneficial. Evidence is not sufficient to determine the best antimicrobial regimen to treat women with intra-amniotic infection because the trials available have investigated a small number of patients and often lack sufficient power for detecting statistical differences among the treatments compared. Nevertheless, the last Cochrane review, from 2014 (50), concluded that the evidence quality ranged from low to very low for the majority of outcomes, following the GRADE approach. The evidence available is too limited for revealing the best antimicrobial regimen for treating patients experiencing chorioamnionitis, whether it is appropriate to continue antibiotics postpartum, and what treatment duration or which antibiotic regimen ought to be used (50).

In contrast to the dilemmas of antibiotic therapy, the mode of delivery is a less problematic question. Namely, if clinical chorioamnionitis is diagnosed, delivery should be considered regardless of the gestational age, but this does not necessarily mean a cesarean delivery is needed (1, 4). It is important to ensure proper labor induction and progression if not contraindicated. Vaginal delivery is generally safer, and cesarean delivery should only be used for standard obstetric indications (4). Venkatesh et al. (46) conducted a large multicenter retrospective cohort study that supports this recommendation, finding that clinical chorioamnionitis is associated with an increased risk of adverse maternal outcomes for women that have a cesarean delivery, regardless of the type and duration of antibiotic therapy, but not for those that have a vaginal delivery (46). The study included 216,467 women without clinical chorioamnionitis and 4,807 women with clinical chorioamnionitis, with 2,794 delivering vaginally and 2,013 undergoing cesarean delivery. The adjusted odds ratio for adverse maternal outcomes was 2.31 (95% CI 1.97–2.71) for cesarean delivery and 1.15 (95% CI 0.93–1.43) for vaginal delivery. Another study by D’Arpe et al., analyzing the causes of peripartum hysterectomy, found that the cause for atony was rarely a peripartum infection (47). However, more research is needed on the relationship between chorioamnionitis and caesarean section. We should also not forget to mention the new SARS-CoV-2 strain. Pregnant women are infected as often as the general population, but a higher proportion of them recover from the infection without symptoms compared to the non-pregnant population. However, does COVID-19 cause chorioamnionitis? Pregnant women belong to a vulnerable population because they have a higher risk of complications due to anatomical, physiological, hormonal, and immunological changes during pregnancy. SARS-CoV-2 enters the cells of the lungs and other organs via the angiotensin-converting enzyme 2 receptor (ACE2 receptor) (48). Binding of the virus to ACE2 causes downregulation of this enzyme, resulting in reduced conversion of angiotensin II to angiotensin. The ACE2 receptor plays an important role in trophoblast proliferation, angiogenesis, and the regulation of arterial blood pressure during pregnancy (49). Down-regulation of ACE2 in the placenta due to SARS-CoV-2 may lead to oxidative stress of the placenta and the release of anti-angiogenic factors, including soluble fms-like tyrosine kinase-1 (sFlt-1) (50), and a decrease in pro-angiogenic factors, leading to features of pre-eclampsia and HELLP syndrome (49). When infected with SARS-CoV-2, symptomatic pregnant women have an increased risk of preterm birth, with delivery before the 37th week of pregnancy, especially iatrogenic preterm birth, due to worsening of the condition of the pregnant woman or the fetus. The increased risk of preterm birth after infection with SARS-CoV-2 is also related to the gestational age at the time of infection and different strains of SARS-CoV-2. SARS-CoV-2 can cause inflammation and damage to the placenta, which in turn increases the risk of developing preeclampsia and stillbirth and thus the risk of premature birth. The large multinational INTERCOVID (51) cohort study assessed maternal and neonatal outcomes in a group of pregnant women with COVID-19 (*n* = 706) compared to a group without COVID-19 (*n* = 1,424) and found that pregnant women with COVID-19 had an increased risk of preterm birth; 83% of preterm births had a medical indication, and the main diagnoses were pre-eclampsia/eclampsia/HELLP (24.7%), small size for gestational age (15.5%), and fetal distress (13.2%). Pregnant women with COVID-19 had fewer incidences of spontaneous onset of labor and more caesarean deliveries. Pregnant women with COVID-19 delivered earlier than non–COVID-19 women after 30 weeks of gestation, with the largest difference before 37 weeks of gestation (51). Much of the initial research on the impact of infection on perinatal outcomes was performed in a group of third-trimester pregnant women, and the question of the impact of infection before and after 20 weeks of gestation was raised early. Badr et al. found that SARS-CoV-2 infection in the late second and early third trimesters increased the risk of adverse obstetric and neonatal outcomes, as well as of delivery before 37 weeks of gestation, whereas there were no statistically significant differences between delivery before 32 weeks of gestation and spontaneous delivery before 37 weeks of gestation (52). In the WAPM study on COVID-19 (53, 54), the multinational retrospective cohort study conclusion was that high-risk pregnancies complicated by SARS-CoV-2 were at higher risk of adverse maternal outcomes than low-risk pregnancies complicated by SARS-CoV-2 infection. Moreover, early gestational age at infection, maternal ventilatory supports, and low birthweight are the main determinants of adverse perinatal outcomes in fetuses with maternal COVID-19 infection. In a retrospective multicenter cohort study by Piekos et al. (55) on whether SARS-CoV-2 infection in unvaccinated pregnant women in the first and second trimesters is a risk factor for preterm birth, it was found that the greatest predictor of the size of the fetus at delivery was the size of the fetus at the time of infection. The highest risk of preterm birth is with infection in the first trimester, and the possible causes are thought to be increased levels of ACE2 receptors in the placenta in early pregnancy (ACE2 levels are almost undetectable at birth) and increased risk of placental infection via viral binding to the ACE2 receptor. This results in poorer placental function and an increased risk of fetal growth restriction and fetal distress (55). It is important to be aware that many of the studies were conducted at the beginning of the pandemic, or during the delta and omicron waves, when vaccination of pregnant women was just beginning. By now, most pregnant women have already been exposed to SARS-CoV-2 and probably have a different immune response to the virus than at the beginning of the pandemic, with a lower risk of a more severe course of the disease and thus a lower risk of preterm birth. However, it is important to bear in mind research showing a significant impact of the virus on placental abruption. A poorly functioning placenta can lead to serious complications of pregnancy, affecting both iatrogenic and spontaneous preterm birth. Nevertheless, there are no data that COVID-19 infection itself causes chorioamnionitis.

A question that arises is what happens with the infected and then healed uterus after the postpartum period, considering that a diagnosis of chorioamnionitis was made at delivery. Does the tissue completely heal, or are there any biochemical or histological signs that can predict any complications with subsequent pregnancies and deliveries? Vimercati et al. (56) studied the distance between the caesarean section scar and vesicovaginal fold on vaginal ultrasound as a predictor for pre-labor risk assessment of uterine rupture, which indicates a possible new diagnostic tool that can be used as an inspiration to look for new non-invasive diagnostic methods of any kind and try to decrease maternal and fetal complications in subsequent pregnancies.

## 7. Conclusion

Chorioamnionitis is an infection that often occurs in puerperae that have given birth prematurely, but it is also not excluded even in term births. Correct and timely identification of chorioamnionitis is important, especially recognition of the significance of laboratory findings and clinical signs, because identifying and implementing recommendations for treatment is essential to effectively reduce maternal and neonatal morbidity and mortality. The purpose of prophylactic antibiotic application at delivery is to prevent early neonatal sepsis caused by GBS, prevent maternal infections (chorioamnionitis and postpartum endometritis), and prolong pregnancy in PPROM before 34 weeks of gestation. Chorioamnionitis cannot be cured by antibiotic therapy alone without delivery, and therefore it is necessary to make a decision according to the guidelines for induction of labor or acceleration of delivery. When a diagnosis is suspected or established, it is therefore necessary to apply broad-spectrum antibiotics according to the protocol used by each country, and to continue with them until delivery; Figure 2. The time that elapses between diagnosing clinical chorioamnionitis and delivery is related to the majority of adverse outcomes for mothers and newborns. Antibiotic preferences may vary based on local policy, clinician experience and knowledge, the bacteria causing the infection, antimicrobial resistance patterns, maternal allergies, and availability of drugs.

## Author contributions

DL: conceptualization and methodology. DL and MB: writing—preparing the original draft. DL, MB, GK, and MD: writing—review and editing. MD and GK: supervision. All authors have read the published version of the manuscript and agreed to it.
